# A retrospective survey of patient experiences during hospital-based isolation for tuberculosis in new York City

**DOI:** 10.1016/j.jctube.2026.100642

**Published:** 2026-08-27

**Authors:** Marco M. Salerno, Emily F. Squire, Anita Khilall, Maksudur Rahman, Sneha Maru, Priscilla Moreira, Celia Quinn, Joan M. Mangan, Joseph Burzynski

**Affiliations:** a Global Tuberculosis Institute, New Jersey Medical School, Rutgers University, Newark, NJ, USA; b Bureau of Tuberculosis Control, New York City Department of Health and Mental Hygiene, Queens, NY, USA; c Public Health Associate Program, National Center for State, Tribal, Local, and Territorial Public Health Infrastructure and Workforce, Centers for Disease Control and Prevention, Atlanta, GA, USA.; d Division of Disease Control, New York City Department of Health and Mental Hygiene, Queens, NY, USA; e Division of State and Local Readiness, Centers for Disease Control and Prevention, Atlanta, GA, USA; f Division of Tuberculosis Elimination, Centers for Disease Control and Prevention, Atlanta, GA, USA

**Keywords:** Isolation, Hospital-based isolation, Tuberculosis, Patient experience, New York City

## Abstract

**Background:**

To prevent tuberculosis (TB) transmission, individuals with presumed or confirmed respiratory TB are isolated until they are no longer considered infectious. Minimal research exists on the impact of prolonged isolation on persons with TB (PWTB). We surveyed PWTB following hospital discharge when hospital-based isolation (HBI) occurred.

**Design:**

From September 2023 to June 2024, adult PWTB who had experienced HBI were invited to participate in a survey at a New York City Health Department Chest-clinic. The survey was administered by project coordinators or self-administered by patients. Survey questions were adapted from validated instruments, and assessed mental adjustments to TB, access to tangible support, and incurred economic costs during HBI.

**Results:**

Fifty PWTB completed the survey. Participants were in HBI for a median of 23 [IQR: 13–38] days. Survey items assessing cognitive adjustment indicate fighting spirit was the most reported (91% average, range 80%–96%) cognitive strategy used. However, patients also reported feeling hopeless and helpless (28% average, range 20%–44%) and using cognitive avoidance strategies (69% average, range 62%–82%) while in HBI. Sixty-two percent of participants reported lacking at least one form of tangible support (e.g., inability to easily find someone to stay with during an emergency). Thirty-seven (74%) participants reported a loss of employment income and thirty-nine (78%) reported difficulties paying for usual expenses.

**Conclusion:**

Participants reported financial, social, and cognitive adjustment challenges during HBI for TB. Pathways to reduce HBI duration and approaches to provide or direct patients to sources of support are needed.

## Highlights

• Financial impacts of hospital-based isolation for tuberculosis.
• Patient experiences during hospital-based isolation for tuberculosis.
• Various coping strategies utilized during hospital-based isolation for tuberculosis.

## 1 Introduction

Tuberculosis (TB) is an airborne disease caused by *Mycobacterium tuberculosis* (*M. tuberculosis*) and is primarily transmitted when a person with active pulmonary disease expels droplet nuclei through activities, such as coughing, talking or singing [1]. Documented TB transmission in hospitals during the late 1980s–1990s led to stricter protocols, requiring patients to be placed in airborne isolation and confined to negative pressure rooms while undergoing diagnostic evaluation and treatment initiation [2].

Although hospitalization for TB is not mandatory in New York City (NYC), most patients are diagnosed and initiate treatment in a hospital setting [3]. Hospital-based isolation (HBI) continues to be used as a public health intervention to separate persons with confirmed or suspected infectious TB from others, until the risk of infectiousness has ended [4]. Patients are often kept in HBI throughout their hospitalization, before being discharged to the community [5].

Each NYC hospital facility has developed criteria for initiating and terminating HBI [1]. The prevailing airborne infection isolation criteria require that individuals with a suspected or confirmed diagnosis of TB are placed in isolation, started on standard therapy, and monitored for improvement demonstrated by sputum smear microscopy or clinical signs and symptoms before hospital discharge [1].

### 1.1 Guidelines

The use of HBI as an intervention to prevent TB transmission is consistent with World Health Organization (WHO) guidance published in 2019, and Centers for Disease Control and Prevention (CDC) guidance published in 2005 [6], [7]. While the WHO guidance notes de-isolation should be based on the reduced likelihood of transmission and the availability of other supportive systems, CDC recommends that persons with infectious TB disease be removed from respiratory isolation restrictions once they have three consecutive negative acid-fast bacilli (AFB) sputum smear results, a minimum of two weeks of multi-drug anti-TB treatment, and clinical improvement [6], [7]. These guidelines have led to prolonged periods of isolation because patients may remain AFB smear-positive despite weeks of treatment; in such cases, the bacteria detected by AFB stain are often not viable and thus not infectious [8], [9].

In contrast, a survey of CDC-supported state and big-city TB programs demonstrated a variety of criteria for discontinuing respiratory isolation in community settings [10]. In 2024, due to concern for the lack of standard practice, the National TB Coalition of America published guidelines for respiratory isolation restrictions in the community [11].

### 1.2 Impacts of HBI on TB patients

Isolation for medical conditions other than TB has been found to negatively impact patients' mental health and financial well-being [12], [13], [14], [15], [16]. It is reasonable to expect persons with TB (PWTB) may experience anxiety, depression, anger and loneliness like patients isolated for other infectious conditions [12].

To inform patient-centered approaches to HBI, we conducted a survey among PWTB receiving care through the NYC Health Department.

## 2 Methods

### 2.1 Objective

Our primary objective was to describe patient perceptions of HBI, evaluate patient cognitive adjustment strategies, and assess economic and social costs associated with prolonged HBI. In addition, we elicited advice from PWTB for others experiencing HBI for TB.

### 2.2 Study design

We recruited eligible participants diagnosed with TB disease using a prospective design at two chest-clinics operated by the NYC Health Department. PWTB were invited to participate in the retrospective survey by project coordinators following clinic appointments. The NYC Health Department Institutional Review Board (IRB) categorized this project (23–060) as research in the expedited category and approved under 45 CFR §46.220(b)(1)(i). CDC IRB implemented an agreement relying on the NYC Health Department IRB.

### 2.3 Study participants

Enrollment began September 14, 2023, and concluded June 13, 2024. We included persons ≥18 years, with laboratory-confirmed, or clinical diagnosis of TB disease, who experienced HBI ≥3 days. We recruited English-, Spanish-, and Mandarin-speaking participants using interpreters and translated surveys. PWTB were excluded if they could not complete a survey in the available languages. PWTB were also excluded if hospital discharge occurred less than one week or more than nine months prior to being asked to participate. Further, PWTB were also excluded if respiratory isolation occurred in a non-healthcare setting or psychiatric care setting, if they were currently incarcerated, or had major psychiatric disorders.

### 2.4 Sampling

Fifty surveys were conducted with a convenience sample of fifty participants based on when eligible individuals were present at the two chest-clinics. Based on patient preference, the survey was administered by project coordinators or self-administered by patients.

### 2.5 Survey methods

To guide development of the survey instrument, a Social Ecological Model was constructed to consider the impact of HBI in the context of personal, interpersonal, health setting, diagnosis and treatment, and socio-economic factors. Based on a literature review and consultation, we identified validated scales to adapt for our survey.

The Mental Adjustment to Stroke Scale, which was adapted from the Mental Adjustment to Cancer Scale, and Mini-Mental Adjustment to Cancer Scale were adapted for this project by replacing the word cancer or stroke with tuberculosis [17], [18], [19]. Adapted items assess patients' feelings of helpless and hopelessness, anxious preoccupation, fatalism, fighting spirit, and avoidance [17], [18], [19]. The HIV Stigma Scale and Short HIV Stigma Scale were adapted to assess TB stigma [20]. Items from the Medical Outcomes Study Social Support Survey were adapted to assess support for PWTB during HBI [21], [22]. The demographic questions in the survey were adapted from the Census Bureau's Pulse Survey [23].

The developed survey was reviewed by TB program managers, physicians, patients, and program evaluators to establish face and content validity. Minor edits were made in response to this review. The survey instrument was then piloted with 1 English and 1 non-English speaking patient and updated further based on feedback.

Following this development phase, the final survey instrument was comprised of 78 items pertaining to five mental adjustment strategies (fighting spirit, fatalism, avoidance, anxiousness, and helplessness/hopelessness), social factors (perceived stigma, financial expenses, and family and relationships' support), and person-centered factors (perceived inter-personal skills of providers, patient-centered communication and services, and perceived quality of care).

### 2.6 Study procedures and data collection

Following outpatient clinic appointments, patients were invited to meet with study coordinators to learn about the study and be screened for eligibility in a private room within the chest-clinic. Eligible patients provided informed consent and then completed the survey. Paper surveys were either self-administered or administered by study coordinators, based on participant preference. Language interpretation services were available for participants who were not comfortable or unable to communicate in English. The surveys were available in English, Spanish, and Mandarin. Survey data were entered into a REDCap database [24], [25] (Version 14.0.11 Vanderbilt University, Nashville, TN, USA) hosted on the NYC Health Department server.

### 2.7 Data sources

Additional data pertaining to participants' clinical and demographic characteristics, social and medical history, and hospitalization information were retrieved from the NYC Health Department's surveillance and case management system (Maven 6.3.2., Conduent Inc., Florham Park, NJ). Information related to attended or missed clinic appointments and directly observed therapy (DOT) visits was retrieved from electronic medical records.

### 2.8 Quantitative analysis

We used Wilcoxon tests to compare continuous variables and Pearson's chi-squared or Fisher's exact tests to compare categorical variables. To determine whether participants were representative of the population of TB patients who underwent HBI and were eligible for the study; we compared the age, sex, race and ethnicity of participants to eligible PWTB who did not complete screening or who elected to not participate in the survey (Table 1).

**Table 1:** Demographics of Patients Not Enrolled and Enrolled, and Time Enrolled Participants Spent in Hospital Based Isolation.

Variable	Overall, *n* = 108	Not Enrolled, *n* = 58	Enrolled, *n* = 50	P-value^1^	Time Enrolled Participants (n = 50) Spent in HBI^2^		
					Less than 30 days, *n* = 24	30 days or more, *n* = 26	P-value^1^
Median days of HBI (IQR)^3^	22 (13,34)	22 (13,31)	23 (13,38)	0.4^5^			
Age^4^	39 (30,63)	46 (30,68)	34 (27,51)	0.02^5^	33 (27,41)	34 (28,62)	0.2^5^
Sex Male	75 (69%)	44 (76%)	31 (62%)	0.12	13 (54%)	18 (69%)	0.3
Female	33 (31%)	14 (24%)	19 (38%)		11 (46%)	8 (31%)	
WHO regions of birth^6^				0.07^7^			0.9^7^
African	1 (1%)	0 (0%)	1 (2%)		0 (0%)	1 (4%)	
Americas	55 (51%)	29 (50%)	26 (52%)		13 (54%)	13 (50%)	
European	2 (2%)	2 (3%)	0 (0%)		0 (0%)	0 (0%)	
South-East Asia	14 (13%)	4 (7%)	10 (20%)		4 (17%)	6 (23%)	
Western Pacific	36 (33%)	23 (40%)	13 (26%)		7 (29%)	6 (23%)	
Site of Disease Pulmonary Involvement	106 (98%)	58 (100%)	48 (96%)	0.2^7^	23 (96%)	25 (96%)	>0.9^7^
Extrapulmonary Only	2 (2%)	0 (0%)	2 (4%)		1 (4%)	1 (4%)	
Ever Sputum Smear Positive	73 (69%)^8^	36 (62%)	37 (77%)	0.1	15 (65%)^9^	22 (88%)^9^	0.06
Ever Sputum Culture Positive	93 (88%)^8^	50 (86%)	43 (90%)	0.6^7^	20 (87%)^9^	23 (92%)^9^	0.7^7^
Drug Susceptibility							
Drug Susceptible	81 (79%)^10^	42 (76%)	39 (81%)	0.5^7^	17 (74%)^11^	22 (88%)^11^	0.3^7^
Drug Resistant	22 (21%)^10^	13 (24%)	9 (19%)		6 (26%)^11^	3 (12%)^11^	
Unknown	1^10^	1	0				
Ever Cavitary CXR^12^	20 (19%)^8^	9 (16%)	11 (22%)	0.4	4 (17%)^9^	7 (27%)^9^	0.4
Educational Attainment Did not graduate high school			13 (26%)		2 (8%)	11 (42%)	0.02^7^
High school graduate/GED			16 (32%)		9 (38%)	7 (27%)	
Some college or 2-yr degree or 4-yr college degree			13 (26%)		6 (25%)	7 (27%)	
More than 4-yr college degree			6 (12%)		5 (21%)	1 (4%)	
Prefer not to answer			2 (4%)		2 (8%)	0 (0%)	

Drug resistance includes other-drug resistant (ODR) and multi-drug resistant (MDR). ODR includes resistance to any first line drug but not both isoniazid (INH) and rifampin (RIF); MDR is resistant to at least INH and RIF.

1 Pearson's Chi-squared test unless otherwise noted.

2 Hospital Based Isolation (HBI).

3 Interquartile Range (IQR).

4 Median (IQR).

5 Wilcoxon rank sum test.

6 World Health Organization (WHO).

7 Fisher's exact test.

8 Among all eligible persons with pulmonary involvement only (n = 106).

9 Among all enrolled persons with pulmonary involvement only (n = 48).

10 Drug susceptibility among all eligible persons with a DST done (n = 104).

11 Drug susceptibility among all enrolled persons with a DST done (n = 48).

12 Chest X-ray (CXR).

Responses to mental adjustment strategies employed during HBI were categorized into a dichotomous variable: Agreement: included `Definitely applies to me` and `Applies to me`, and Disagreement included: `Does not apply to me` and `Definitely does not apply to me’.

Additionally, responses to the support participants received from others were categorized into a dichotomous variable: True reflected support and included ‘Definitely true’ and ‘Probably true’ and False reflected little to no support and included: ‘Definitely false’ and ‘Probably false’. Two items required reverse coding.

Responses to the stigma felt while in HBI were categorized into a dichotomous variable: Agreement included ‘Strongly agree’ and ‘Agree’, and Disagreement included `Strongly disagree` and ‘Disagree’. Patients who responded in agreement were categorized as perceiving stigma.

We also compared participants who spent more time (≥30 days) versus participants who spent less time (<30 days) in HBI. We utilized thirty days as the cut-off to assess the impact of prolonged isolation based on previous programmatic evaluations.

### 2.9 Qualitative analysis

Investigators discussed the major themes in participant responses to open-ended questions asking about their advice for other PWTB in HBI, and produced a codebook based on initially identified themes. Three investigators individually coded participants' responses using the codebook and attained an average inter-coder agreement score of 78%. Investigators refined the initial themes, re-coded the data and arrived at a 100% inter-coder agreement score. A fourth investigator performed a final quality control review.

### 2.10 Software

R (R version 4.3.2) was used for cleaning, analyzing, and presenting data [26].

## 3 Results

### 3.1 Eligibility

Between September 11, 2023, and June 13, 2024, 361 PWTB were assessed for survey participation at the chest-clinics. Among this group, 253 (70%) were ineligible. The top three reasons were: 105 (42%) were not hospitalized at the time of diagnosis, 36 (14%) had undergone HBI more than 9 months prior to survey implementation, and 30 (12%) preferred a language unavailable through this study.

### 3.2 Enrolled participants

Of the remaining 108 eligible patients, 77 (71%) were screened. Fifty patients consented to enrollment and fifty completed the survey. Enrolled participants were younger (median age: 34 vs 61, *p*-value: 0.02) compared to eligible patients who declined participation (Table 1). Enrolled participants were mostly non-US born (92%), nearly two-thirds (62%) were male, most had drug-susceptible TB (81%, 39/48), with pulmonary involvement (96%), and were ever sputum smear positive (77%, 37/48). One participant had rifampicin (RIF)-resistant TB, and one had multi-drug resistant (MDR-TB). Among the 48 participants who reported educational attainment, 13 (27%) had not completed high school and 16 (32%) were high-school graduates.

### 3.3 Time in hospital-based isolation

Participants were in HBI for a median of 23 (IQR 13, 38) days. Sputum smear positive participants (*n* = 37) were in HBI for more median days (32 vs 14) compared to sputum-smear negative participants (*n* = 11) (p-value: 0.02). Age, clinical characteristics, and occupational status at the time of survey were not statistically significant. However, participants with greater educational attainment spent fewer median days in HBI. (Table 1).

### 3.4 Financial loss

Most participants reported financial challenges or income loss while in HBI and at the time of the survey: 74% reported a loss of employment income while in HBI, 78% reported difficulties paying for usual expenses during HBI (Table 2). Of the 39 participants who reported difficulties with their household paying usual expenses, 33 (85%) reported income loss while in HBI.

**Table 2:** Reported Finances Among Enrolled Participants.

Variable	n=50^1^
Participant reported a loss of employment income during hospital-based isolation	37 (74%)
Difficulty paying for usual expenses during hospital-based isolation^2^	
Difficult	39 (78%)
Not difficult	11 (22%)
Is household currently caught up on rent/mortgage?^3^	
Yes	26 (57%)
No	14 (28%)
Not sure	6 (12%)
Not applicable	3 (6%)
Unknown / Did not answer	1 (2%)
Occupation at time of diagnosis^4^	
Employed	31 (62%)
Unemployed or Not Seeking	18 (36%)
Unknown	1 (2%)
Occupation at time of survey^5^	
Employed	15 (30%)
Unemployed or Not Seeking	28 (56%)
Other	5 (10%)
Prefer not to answer	2 (4%)

1 n (%).

2 Usual expenses included but were not limited to food, rent or mortgage, car payments, and medical expenses. Difficult includes participants who selected ‘a little difficult’, ‘somewhat difficult’, or ‘very difficult’.

3 Currently refers to at the time of the survey.

4 Employed includes participants who self-reported being employed to case management at time of diagnosis. Unemployed or Not Seeking includes participants who self-reported as ‘disabled’ (n = 2), ‘homemaker’ (n = 1), ‘retired’ (n = 4), ‘student’ (n = 1), or ‘unemployed’ (n = 20) to case management at time of diagnosis.

5 Employed includes participants who self-reported as ‘employed full time’ or ‘employed part time’. Unemployed or Not Seeking includes participants who self-reported as ‘disabled’, ‘homemaker’, ‘retired’, ‘student’, or ‘unemployed’.

Participants who reported losing employment income in isolation (*n* = 37) were in HBI for more median days (25 vs 13, p-value: 0.03) than participants who reported no employment income loss (*n* = 13).

### 3.5 Stigma and social support

With respect to stigma related to their TB diagnosis: 30% (15/50) of participants reported ‘I tried to keep my TB a secret from others’, 58% (29/50) reported ‘I was very careful who I told that I had TB’, and 50% (25/50) reported ‘I worried that people who know I have TB will tell others’. Participants who reported stigma were also in the hospital for greater median days. Participants who reported trying to keep their TB a secret from others (*n* = 15) spent a similar number of days in HBI (32 vs 21; p-value: 0.30) than those that did not report trying to keep their TB a secret (*n* = 24) and those who selected a neutral response (*n* = 11) (32 vs 18; p-value: 0.40).

Participants reported varying levels of social support (Table 3). Participants who had someone they could call for help with meals (*n* = 44) spent fewer median days in HBI (21 vs. 55; p-value: 0.01) compared to those that did not report this (*n* = 6).

**Table 3:** Comparison of Median Days Spent in Hospital-based Isolation Among Participants Who Did and Did Not Report Presence of Tangible Levels of Various Types of Social Support.

Variable	Tangible Support Median (IQR), n	No Support Median (IQR), n	P-value^1^
If sick, I could easily find someone to help with daily chores	23 (11, 35), *n* = 42	36 (17,53), *n* = 8	0.14
If I needed to stay in bed, I could find someone to help	22 (12,34), *n* = 37	37 (17, 51), *n* = 13	0.10
If I needed help with meals, there would be someone I could call	21 (12, 35), *n* = 44	55 (34,62), *n* = 6	0.01
If I needed someone to take me to the doctor, I could find someone	23 (13, 40), *n* = 34	23 (15,37), *n* = 16	0.8
If I needed a place to stay for a week because of an emergency, I could find someone to put me up	22 (12,35), *n* = 31	23 (15,49), *n* = 19	0.3

1 Wilcoxon rank sum test.

### 3.6 Coping: mental adjustment strategies

**Fig. 1:**
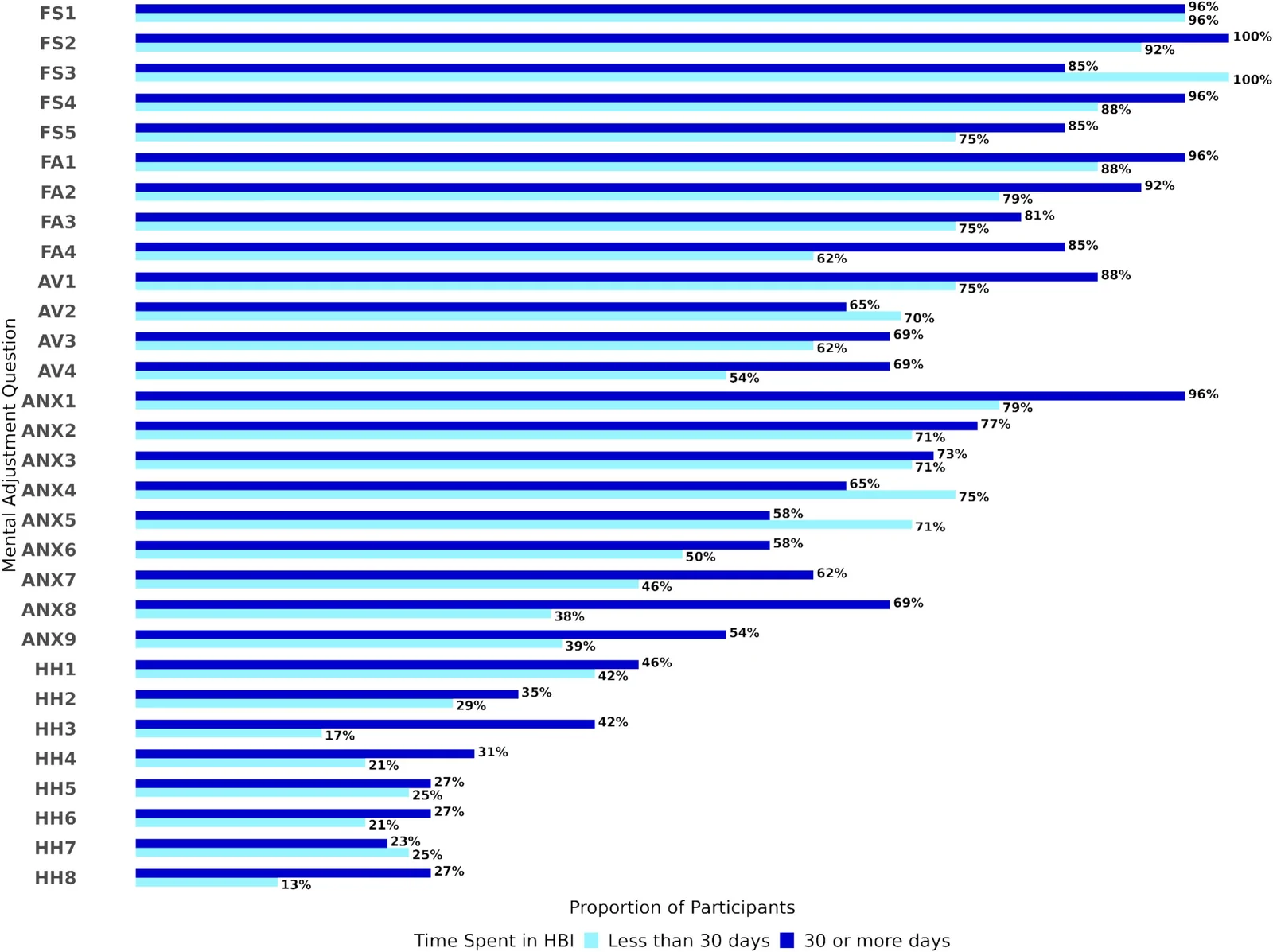
Percentage of Participants That Affirmed Using Various Mental Adjustment Strategies During Hospital-based Isolation (*n* = 50). Key During HBI… Fighting Spirit. FS1=I firmly believed that I would get better. FS2=I was determined to beat TB. FS3=I tried to fight the illness. FS4=I was very optimistic. FS5=I saw my illness as a challenge. Fatalism. FA1 = I realized how precious life is and made the best of it. FA2 = At that moment, I took one day at a time. FA3 = I put myself in the hands of God. FA4 = I counted my blessings. Avoidance. AV1 = I made a positive effort to not think about my illness. AV2* = I distracted myself when thoughts about my illness came into my head. AV3 = Not thinking about TB helped me to cope. AV4 = I deliberately pushed all thoughts of TB out of my mind. Anxious. ANX1 = I was a little frightened. ANX2 = I had difficulty believing what was happening to me. ANX3 = I was upset about having TB. ANX4 = It was upsetting. ANX5 = I was anxious. ANX6 = I did not really believe that I had TB. ANX7 = I felt very angry about what was happening to me. ANX8 = I suffered great anxiety about it. ANX9* = I worried about my TB getting worse. Helpless and Hopeless. HH1=I felt discouraged. HH2=I felt that there was nothing I could do to help myself. HH3=I could not handle it. HH4=I could not cope. HH5=I was not very hopeful about the future. HH6=I felt completely at a loss about what to do. HH7=I thought it was the end of the world. HH8*^=I felt like giving up. * = Questions had 49 respondents. ^ = Participants that reported ‘I felt like giving up’ had similar treatment outcomes when compared to participants that did not report this. All participants that reported ‘I felt like giving up’ completed treatment.

‘Fighting spirit’ concepts were the most reported cognitive adjustment strategies (91% average, range 80%–96%; Fig. 1) among participants. Most (96%) agreed that during HBI, ‘I was determined to beat TB’, including all participants (*n* = 26) who spent ≥30 days in HBI, compared with 22 of 24 participants who spent <30 days in HBI (p-value: 0.2). However, the forty-six participants who reported, `I tried to fight the illness`, spent fewer median days in HBI (22 vs. 50, p-value: 0.01) than those that did not report this.

Additionally, many participants engaged in fatalistic thinking (82% average, range: 74%–92%), cognitive avoidance (69% average, range: 62%–82%), and anxious preoccupation (64% average, range: 47%–88%). Of the 27 (54%) participants who answered affirmatively to ‘I suffered great anxiety about it’, 18 (67%) spent ≥30 days in HBI, compared to 9 (33%) that spent <30 days in HBI (p-value: 0.03).

Fewer participants reported concepts of feeling helpless or hopeless (28% average, range: 20%–44%) than other mental adjustment domains. Of the 15 participants who indicated `I could not handle it` applied to them while in HBI, 11 (73%) spent ≥30 days in HBI, compared to 4 (27%) that spent <30 days in HBI (p-value: 0.05). Nonetheless, one fifth (10/49) of the participants felt like giving up while in isolation.

### 3.7 Clinical outcomes

Most participants completed treatment (96%), one participant moved during treatment, and one was lost during follow-up (Table 4). Participants had a median clinic appointment adherence of 75% (range: 36%–100%) and DOT adherence of 77% (range: 19%–94%). Participants who spent ≥30 days in HBI (*n* = 26) had a similar median clinic appointment adherence (74% vs 78%, p-value: >0.9) compared to participants who spent <30 days in HBI (*n* = 24).

**Table 4:** Clinical Outcomes and Number of Days Spent in Hospital-based Isolation.

Variable	Overall, *n* = 50	Less than 30 days, *n* = 24	30 or more days, *n* = 26	P-value^1^
Final Disposition Completed treatment Lost Moved	48 (96%) 1 (2%) 1 (2%)	22 (92%) 1 (4%) 1 (4%)	26 (100%) 0 (0%) 0 (0%)	0.2^2^
Clinic Appointment Adherence^3^, ^4^	75% (67%, 88%)	78% (66%, 89%)	74% (67%, 86%)	> 0.9
DOT Adherence^3^, ^5^	77% (61%, 89%)	79% (64%, 89%)	77% (61%, 88%)	>0.9

1 Wilcoxon rank sum test, unless otherwise noted.

2 Fisher's exact test.

3 Median (interquartile range).

4 Clinic appointment adherence is the percentage of appointments that a survey participant attended with a doctor or nurse out of the total scheduled appointments, analyzed as a continuous variable.

5 Directly Observed Therapy (DOT) adherence is the proportion of scheduled DOT appointments that a survey participant complies with under New York City DOT requirements Analyzed as a continuous variable among participants ever on DOT (n = 47).

### 3.8 Advice

The advice provided by most participants for others experiencing HBI included ‘trusting the providers’ treatment plan’ (*n* = 28), ‘being positive’ (*n* = 22), and ‘seeking out a past time or hobby’ (*n* = 12). Additional themes were: ‘being informed of treatment’, ‘utilizing social skills', and ‘using coping mechanisms.’ Suggested coping mechanisms included expressing faith or religion (*n* = 11), remaining positive (*n* = 22), acknowledging HBI is a difficult experience (*n* = 7), and accessing social support (*n* = 7).

## 4 Discussion

In our prospective cohort, we observed perceptions of stigma, differing coping strategies, and prevalent financial impacts of HBI.

Among our participants, most remained in HBI for greater than three weeks (median 23 days), and over a quarter in HBI for greater than one month (75th percentile 38 days), in NYC. Smear-negative patients (*n* = 13), and patients with extrapulmonary manifestations (*n* = 2) who are generally considered non-infectious endured HBI an average of 16 and 10 days respectively. These individuals could be spared social and economic impacts associated with HBI.

Among the study participants, loss of employment income and financial difficulties were common. Median time in HBI was greater among individuals who experienced loss of employment income. Few investigators have demonstrated HBI's financial and social impacts on PWTB. [27], [28], [29], [30] Direct financial costs include physician fees and hospital fees. Indirect costs may include absence of or reduced income for PWTB who are unable to work and those financially dependent on the patients [27], [30].

In our study, most participants reported mental adjustment strategies of fighting spirit, anxiousness, fatalistic thoughts, and cognitive avoidance. Prolonged HBI may worsen psychological responses during this crucial period of TB care. Though generally less reported, 28% of participants feeling helpless or hopeless is cause for concern. The psychological impacts of prolonged isolation reported by our participants are consistent with reports in the literature [6], [12], [13], [14], [15], [16], [17], [18], [19], [30], [31], [32]. The WHO reports that persons isolated for extended periods experience greater anxiety, depression, anger, and feelings of imprisonment [6]. A systematic review of persons isolated for antimicrobial resistance reported negative impact on patients' moods, including anxiety, depression, fear, loneliness, and hostility [12]. Additionally, individuals isolated for MERS experienced anxiety and anger with mental health effects lasting four to six months post discharge [16]. These data, in conjunction with the hopelessness reported by participants in this study indicate a need for TB Programs to anticipate, screen for, and provide support to patients with poor psychosocial wellbeing both during and after HBI to optimize treatment outcomes.

Stigma has been well-documented as a social impact experienced by PWTB. Persons in isolation also report stigma and psychosocial concerns related to separation from family and society [27], [29], [30]. Our results indicate that HBI duration is associated with perceptions of stigma and lack of social support.

In our small study, with most patients having favorable outcomes, we were unable to detect any significant relationship between clinical outcomes of appointment adherence, DOT adherence, treatment completion, and HBI duration (< 30 days or ≥ 30 days). This may reflect the strength of the NYC TB Program and its programmatic support, such as case management throughout the entirety of treatment. If this study was replicated on a larger scale or in another setting, this finding may differ. In the literature, findings on the relationship between clinical outcomes and HBI are inconsistent [12], [33].

Healthcare personnel and future patients may use the participants' advice, such as trusting the treatment plan, being positive and informed of treatment, as an opportunity to improve patients' HBI experiences.

Recognizing the challenges noted above, updates to NYC Health Department Isolation Recommendations aim to shorten the duration of hospital stays, by evaluating individuals' living and working environments. [1], [4] Additional assessments, conducted when a person is placed in HBI, could direct patients to sources of support to help alleviate psychological and social challenges of HBI.

## 5 Limitations

In NYC, the chest-clinics only see about 50% of PWTB [3]. Thus, the participants in this small study are not representative of all NYC PWTB. Chest-clinics provide free TB treatment to all patients, regardless of ability to pay. As the study population is accessing care provided at no cost, it is essential to understand their vulnerabilities, the impacts of HBI, and consider whether HBI helps address gaps or allows time to arrange services outside the hospital. Study data do not address these situations. The survey was only administered at outpatient chest-clinics in English, Mandarin, or Spanish, further limiting external validity. Additionally, since the survey was administered at least a week after hospital discharge, there may have been recall bias. Project coordinators were trained in strategies to help PWTB with recall.

## 6 Conclusion

HBI has a substantial financial, social, and behavioral impact on PWTB. Strategies to mitigate the impact of HBI are needed.

## Statement for studies in humans/animals

Following outpatient clinic appointments, patients were invited to meet with study coordinators to learn about the study and be screened for eligibility in a private room within the chest-clinic. Eligible patients provided informed consent and then completed the survey. Paper surveys were either self-administered or administered by study coordinators, based on participant preference. Language interpretation services were available for participants who were not comfortable or unable to communicate in English.

## Data statement

Individual-level surveillance data are not publicly available because they contain confidential public health information. Aggregate, nonidentifying data may be available from the corresponding author and NYC DOHMH subject to applicable approvals.

## CRediT authorship contribution statement

**Marco M. Salerno:** Writing – review & editing, Writing – original draft, Visualization, Supervision, Project administration, Methodology, Formal analysis, Data curation, Conceptualization. **Emily F. Squire:** Writing – review & editing, Writing – original draft, Visualization, Project administration, Investigation, Formal analysis. **Anita Khilall:** Supervision, Resources, Project administration, Methodology. **Maksudur Rahman:** Project administration, Investigation. **Sneha Maru:** Methodology, Formal analysis. **Priscilla Moreira:** Resources, Methodology. **Celia Quinn:** Writing – review & editing, Supervision. **Joan M. Mangan:** Writing – review & editing, Writing – original draft, Supervision, Project administration, Methodology, Conceptualization. **Joseph Burzynski:** Writing – review & editing, Writing – original draft, Supervision, Formal analysis, Conceptualization.

## Ethics declaration

### Informed consent and patient details

Written informed consent to take part in the study and to publish the article has been obtained from all participants or their legal representatives. The privacy rights of participants have been observed.

### Studies in Human

This study was performed in compliance with relevant laws, regulatory frameworks and guidelines where the research took place.

This study was approved by the New York City Health Department Institutional Review Board.

(Approval No. 23–060)

## Funding

This research did not receive any specific grant from funding agencies in the public, commercial, or not-for-profit sectors.

## Declaration of competing interest

The authors declare that they have no known competing financial interests or personal relationships that could have appeared to influence the work reported in this paper.

The findings and conclusions in this report are those of the authors and do not necessarily represent the official position of the Centers for Disease Control and Prevention, the Department of Health and Human Services, or the U.S. Government.
