# Supplementary material for: A retrospective survey of patient experiences during hospital-based isolation for tuberculosis in new York City
Source: J Clin Tuberc Other Mycobact Dis. 2026 Aug 27;45:100642. doi: 10.1016/j.jctube.2026.100642 (PMC13571502; doi:10.1016/j.jctube.2026.100642)
Supplement: Supplementary file 1 [file mmc1.docx]

**Supplement A**

Under New York City (NYC) Department of Health recommendations, a person with potentially infectious tuberculosis (TB) should be placed in an airborne infection isolation room but isolation is not required for persons with TB that does not involve the respiratory system.^1^ During hospital based isolation (HBI), patients are restricted to the room except when a diagnostic exam cannot be done in the room.^1^ In NYC, every facility has its own procedures for initiating and discontinuing HBI and hospitals in NYC are required to notify NYC Health Department prior to discharging infectious TB patients.^1^ At the time of data collection, persons with infectious TB were generally in HBI until they completed two weeks of anti-tuberculosis treatment and/or had three consecutive negative smears. However, in 2025 the NYC Health Department updated isolation recommendations for individuals with respiratory TB to align with the National Tuberculosis Coalition of America (NTCA) guidelines for community settings. Once a person with TB is discharged from the hospital, they have the option to follow up with a NYC TB chest-clinic or a non-Health Department provider.

^1. New York City Department of Health and Mental Hygiene. Tuberculosis Clinical Policies and Program Manual^*^.^* [^https://www.nyc.gov/site/doh/providers/health-topics/tb-hosp-manual.page Published December 2025^](https://www.nyc.gov/site/doh/providers/health-topics/tb-hosp-manual.page%20Published%20December%202025)^.^
